# GGA-MLP: A Greedy Genetic Algorithm to Optimize Weights and Biases in Multilayer Perceptron

**DOI:** 10.1155/2022/4036035

**Published:** 2022-02-24

**Authors:** Priti Bansal, Rishabh Lamba, Vaibhav Jain, Tanmay Jain, Sanchit Shokeen, Sumit Kumar, Pradeep Kumar Singh, Baseem Khan

**Affiliations:** ^1^Department of Information Technology, Netaji Subhas University of Technology, Dwarka, New Delhi, India; ^2^Department of Computer Science and Engineering, Amity School of Engineering and Technology, Amity University Uttar Pradesh, Noida, India; ^3^Department of Computer Science, KIET Group of Institutions, Delhi-NCR, Ghaziabad, India; ^4^Hawassa University, Hawassa, Ethiopia

## Abstract

The task of designing an Artificial Neural Network (ANN) can be thought of as an optimization problem that involves many parameters whose optimal value needs to be computed in order to improve the classification accuracy of an ANN. Two of the major parameters that need to be determined during the design of an ANN are weights and biases. Various gradient-based optimization algorithms have been proposed by researchers in the past to generate an optimal set of weights and biases. However, due to the tendency of gradient-based algorithms to get trapped in local minima, researchers have started exploring metaheuristic algorithms as an alternative to the conventional techniques. In this paper, we propose the GGA-MLP (Greedy Genetic Algorithm-Multilayer Perceptron) approach, a learning algorithm, to generate an optimal set of weights and biases in multilayer perceptron (MLP) using a greedy genetic algorithm. The proposed approach increases the performance of the traditional genetic algorithm (GA) by using a greedy approach to generate the initial population as well as to perform crossover and mutation. To evaluate the performance of GGA-MLP in classifying nonlinear input patterns, we perform experiments on datasets of varying complexities taken from the University of California, Irvine (UCI) repository. The experimental results of GGA-MLP are compared with the existing state-of-the-art techniques in terms of classification accuracy. The results show that the performance of GGA-MLP is better than or comparable to the existing state-of-the-art techniques.

## 1. Introduction

Artificial Neural networks (ANNs) are computing models inspired by the biological nervous system. An ANN consists of an interconnected network of nodes called artificial neurons which are organized in the form of layers, namely, input layer, hidden layers, and output layer [1]. A set of synaptic weights is used to interconnect the nodes that form these layers. ANNs have been applied to a broad range of problems like classification, regression, prediction, pattern recognition, and disease diagnosis [2–6]. Classification is one of the important areas of research in the field of data science. Many classification models exist, out of which ANNs are among the most widely used models.

In this paper, our focus is on multilayer perceptron (MLP) which is a multilayer feedforward neural network. Classification using MLP is basically a two-step process. The first step is the learning (training) phase in which a classifier is built to describe a predetermined set of data classes for a given dataset (training data). In the second step, the model which has been built in the training phase is used for the classification of the unclassified data (test data) for estimating the accuracy of the classifier. During the learning phase, MLP learns by adjusting synaptic weights and biases iteratively in an attempt to correctly predict the class labels of the input data. The process of weight and bias update continues until the acquired knowledge is sufficient and the network reaches a specified level of accuracy; i.e., a predefined error measure is minimized, or the maximum number of epochs is reached [7]. After the completion of the learning phase, it is mandatory to assess the performance of MLP, i.e., its generalization and predictive capabilities, using samples of data (test data) that are different from those used during the training phase for the given dataset. To achieve generalization, MLPs need to avoid the issues of both underfitting and overfitting during the training phase. To achieve the best results, it is therefore required that the number of training patterns should be sufficiently larger than the total number of connections in the neural network. The performance of MLP is highly dependent on the learning method used to train it during the training phase. Several learning algorithms exist in the literature with the aim of finding an optimal MLP. These learning algorithms can be broadly classified into three categories, namely, conventional methods [8–12], metaheuristic-based methods [13–37], and hybrid methods [20, 38–44].

Despite the existence of a large number of learning algorithms, researchers continue to apply new optimization techniques like multimean particle swarm optimization (MMPSO) [28], whale optimization algorithm (WOA) [23], multiverse optimizer (MVO) [34], grasshopper optimization algorithm (GOA) [35], and firefly algorithm [36] to generate an optimal set of synaptic weights in an attempt to further improve the accuracy and performance of MLP. As stated in No-Free-Lunch (NFL) theorem [45], there is no optimization technique that solves all optimization problems. It is quite possible that an existing learning algorithm may train an MLP well for some datasets while it fails to do the same for some other datasets. This makes the field of generating optimal connection weights a dynamic research area. This is the main motivation behind the work presented in this paper, in which we propose a hybrid learning algorithm to train MLP.

GA is an evolutionary algorithm (EA) and is one of the most widely investigated algorithms among the metaheuristic algorithms in designing neural networks. Over the years, GA and its variants have been successfully applied in several domains for ANN weight [13–20], topology [46–48], and feature set optimization [49, 50], as well as parameter tuning [51, 52]. A comprehensive review of optimization of neural networks using GA can be found in [53]. The efficiency, effectiveness, and ease of use of GA motivated us to further improve the performance of GA in optimizing weights of MLP by integrating greedy techniques with GA. The proposed algorithm Greedy Genetic Algorithm–Multilayer Perceptron (GGA-MLP) improves the performance of traditional GA by using a greedy approach to generate the initial population as well as to perform crossover and mutation. Some of the application areas of the proposed work are disease identification, e-mail spam identification, prediction of the stock market, and fruit classification. The main challenge with the proposed approach is that it may not work well with some of the datasets, as stated by No-Free-Lunch (NFL) theorem [45] mentioned above. Finally, the performance of GGA-MLP is compared with various classifiers as well as the existing state-of-the-art metaheuristic algorithms for training MLP. The key contributions of this paper are as follows:

1. A hybrid learning algorithm, GGA-MLP, that integrates greedy techniques with GA is proposed to train MLP
2. GGA-MLP is evaluated and compared with existing state-of-the-art algorithms on 10 datasets of different complexities

The paper is organized as follows. Related work is presented in Section 2. A brief overview of GA is given in Section 3. In Section 4, the proposed GGA-MLP for optimization of MLP weights and biases is presented. In Section 5, experiments conducted to evaluate the effectiveness of GGA-MLP are presented, and results are discussed. Finally, the conclusion and future work are discussed in Section 6.

## 2. Related Work

In conventional methods, backpropagation (BP) is the most widely used algorithm to train multilayer feedforward networks (MLFFNs). BP uses a gradient descent rule that tries to minimize the error of the network by moving in a direction opposite to that of the gradient of the error function. However, BP has certain limitations. It has a tendency to converge toward the local optima, as it is good only at exploiting the current solution, which may result in unsatisfactory classification accuracies. It also has slow convergence as well as scaling problems [54]. To overcome these problems, many improvements of BP such as quickprop [8], RPROP [9], and improved BP [10] have been proposed by researchers in the past. Besides, conjugate gradient methods [11] and other derivative-based conventional methods such as Levenberg–Marquardt method [12] are also used for weight optimization, but sometimes these methods can be expensive. Conventional methods are computationally faster as compared to their metaheuristic counterparts because they operate on a single solution; however, they have certain limitations as discussed above.

Due to the global search capabilities of metaheuristic algorithms, they are being widely used by researchers to generate optimal weights and biases in MLP. In [13–21], GA was applied to train MLP, and its performance was compared to BP. Valian et al. [22] proposed an improved cuckoo search (ICS) to train MLFNN. Unlike cuckoo search, the proposed ICS tunes the parameters of CS. The performances of ICS and CS are compared on two datasets. A number of approaches have been proposed by researchers to train MLFNN using differential evolution (DE) and evolutionary strategies [23–26]. Apart from EA, bioinspired algorithms and their variants are proposed and used by researchers to generate optimal connection weights in MLFNN. Karaboga et al. [27] applied artificial bee colony (ABC) algorithm to train MLFNN and compared the performance of ABC with that of GA. In [28], multimean particle swarm optimization (MMPSO) is proposed by the authors to generate optimal connection weights of MLFNN. MMPSO is derived from PSO, and unlike PSO it uses multiple swarms. The performance of MMPSO is compared with PSO on 10 datasets, and the results prove the effectiveness of MMPSO. In [29], krill herd algorithm (KHA) is applied to train ANN and is compared with BP, GA, and harmony search (HS). Bolaji et al. [30] and Kattan et al. [31] used fireworks algorithm (FWA) and HS, respectively, to train ANN. Mirjalili [32] applied gray wolf optimizer (GWO) to train MLP, and the comparison results on 8 datasets show the GWO algorithm's capability of avoiding local optima. Aljarah et al. [33] applied a whale optimization algorithm (WOA) to generate an optimal set of connection weights in MLP. The performance of the proposed WOA-based trainer is evaluated on 20 datasets by comparing it with the trainers obtained using ant colony optimization (ACO), GA, PSO, DE, ES, population-based incremental learning (PBIL), and BP. The results indicate that WOA-based trainer avoids premature convergence and generates the best optimal weights in most of the cases for binary pattern classification. In [34], nature-inspired multiverse optimizer is used to train MLP. Heidari et al. [35], proposed GOAMLP that uses GOA to train single hidden layer MLP and is applied on five datasets. When compared with state-of-the-art algorithms, MLP trained using GOAMLP resulted in improved classification accuracy. Elakkiya and Selvakumar [36] used enhanced step size firefly algorithm to generate optimal weights of feedforward neural network for spam detection. In [37], adaptive GA has been proposed for weight optimization of BPNN for capacitive accelerometers. The optimized BPNN is used in the capacitive accelerometer.

Sometimes, metaheuristic algorithms suffer from premature convergence. To overcome the problems faced by conventional methods and metaheuristic algorithms, hybrid approaches were proposed by researchers. In [38, 39], GA and PSO, respectively, have been combined with BP, which helped in fast convergence and avoidance of getting trapped in local optima. In [40], a hybrid approach that combines PSO and gravitational search algorithm is presented to train feedforward networks. In [41], a hybrid training algorithm, LPSONS, is proposed to train feedforward neural networks. It combines the velocity operator of PSO with Mantegna Levy distribution to increase the diversity of the population. To avoid local optima and premature convergence, Mantegna Levy distribution is further combined with neighborhood search. In [20], an improved GA coupled with BP neural network (IGA-BPNN) is proposed to improve the forecast performance of ANN. This model uses improved genetic adaptive strategies to avoid getting stuck in local optima. The experimental results show that IGA-BPNN performs better than traditional GA-BPNN. In [42], a hybrid algorithm, namely, constriction coefficient-based particle swarm optimization and gravitational search algorithm (CPSOGSA), is proposed to train MLP. It helps to avoid premature convergence and getting stuck in local optima problems of MLP. In [43], an optimized adaptive GA in the backpropagation neural network (OAGA-BPNN) is proposed to optimize BPNN for traffic flow prediction. In [44], a hybrid grasshopper and new cat swarm optimization algorithm was proposed for feature selection and weight and architecture optimization of MLP. In a similar way, other optimization approaches are also discussed by various researchers like MLP-LOA [55], improved teaching learning (TLB), and cat swarm optimization to get better results in respect of similar applications [56, 57].

## 3. Genetic Algorithm

Genetic algorithm (GA) is a metaheuristic algorithm proposed by Holland [58]. This algorithm imitates the process of natural selection where the chances of survival of fitter individuals are more as compared to other individuals in a competing environment. It is a global search technique characterized by evolution in every generation. GA starts with a randomly generated initial population of chromosomes where each chromosome represents a possible solution to the given problem. Each chromosome is associated with a ﬁtness value that is a measure of how good a solution is for the given problem. In each generation, the population evolves toward better fitness using evolutionary operators such as selection, crossover, and mutation. This process continues until a solution is found or the maximum number of iterations is reached.

## 4. Proposed Model: GGA-MLP

In this section, we present our proposed approach GGA-MLP which applies a greedy GA to generate an optimal set of synaptic weights and biases of MLP, keeping the architecture and activation function fixed. The various steps of GGA-MLP are explained below.

### 4.1. Representation of Candidate Solutions and Fitness Function

**Figure 1:**
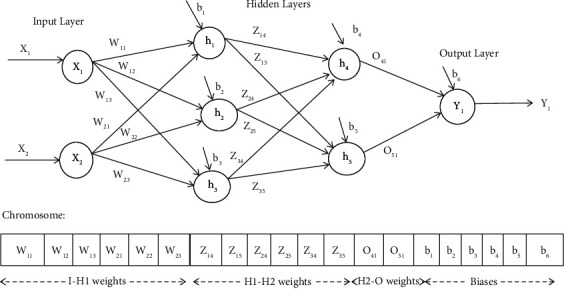
Chromosome encoding of MLP for weight and bias optimization.

An important aspect that needs to be considered during the design of GGA-MLP is the representation of the possible solutions in the search space in the form of chromosomes and the encoding scheme used to encode the chromosomes. In GGA-MLP, each chromosome represents a candidate MLP. A chromosome is basically divided into different segments, where each segment contains the encoded weights between two layers (input-hidden, hidden-hidden (if any), hidden-output) and the last segment contains the encoded bias values for the MLP. Chromosome encoding for an MLP having two hidden layers is shown in Figure 1. However, the length of the chromosome can easily be changed to train MLP having one or more hidden layers. A real value encoding scheme is used to encode the chromosomes.

As it is clear from Figure 1, if there are *n* input nodes, *m* hidden layers with *h*_1_,  *h*_2_,   … .., *h*_*m*_ hidden nodes in each hidden layer, and *k* output nodes, then the length of the chromosome will be calculated using

(1) $$\begin{array}{c} C_{length}=\left(n\;\times h_{1}\right)+\left(\sum_{i=1}^{m-1}h_{i}\times h_{i+1}\right)+\;\left(h_{m}\times k\right)+\sum_{i=1}^{m}h_{i}+k\text{.} \end{array}$$

Each chromosome in the population is represented by *MLP*_*j*_*|* 1 ≤ *j* ≤ *PS* where PS is the population size. Each *MLP*_*j*_*|* 1 ≤ *j* ≤ *PS* in the population is associated with a fitness value which is the measure of its quality. In our case, mean square error (MSE) is chosen as the fitness function. To calculate the fitness of an MLP, the training data sample is made to run on it and the mean square error value is calculated using

(2) $$\begin{array}{c} Fitness\left(MLP\right)=MSE=\;\frac{1}{n}\;\sum_{k=1}^{n}{\left(y_{k}-\;{\hat{y}}_{k}\right)}^{2}\text{,} \end{array}$$

where *y*_*k*_ is the actual output, ${\hat{y}}_{k}$ is the predicted output, and *n* is the number of samples in the training dataset. This process is repeated for each *MLP*_*j*_. The goal of GGA is to find an *MLP* that minimizes the objective function *f* *|* *f* :  *MLP*_*j*_⟶*R*^+^, where R^+^ represents a set of real numbers. The objective function *f* can be calculated using (3), and it tells us about the quality of the solution.

(3) $$\begin{array}{c} f\left(MLP_{j}\right)=fitness\left(MLP_{j}\right)\text{.} \end{array}$$

Now, GGA tries to find the best MLP that minimizes the objective function *f* as shown in

(4) $$\begin{array}{c} MLP_{best\;}=MLP_{l}|\;f\left(MLP_{l}\right)<f\left(MLP_{j}\right)\;\forall j\wedge j\neq l\text{.} \end{array}$$

### 4.2. Generation of Initial Population

In evolutionary algorithms (EAs), the initial population plays a major role in determining the quality of the final solution as well as the convergence speed [59]. Several population initialization methods exist in literature, but in most cases, the initial population is generated randomly. However, due to the dependence of the final solution's quality on the initial population, GGA uses a greedy population initialization method that uses domain-specific knowledge to generate good quality MLPs (chromosomes). Initially, the synaptic weights and biases are chosen randomly in the interval [−2, 2]. After this, GGA analyzes the features of the dataset on which the MLP needs to be trained. In most cases, it has been observed that certain features contribute more than others to determining the correct class of the input pattern. GGA exploits this property of the dataset and finds important features using domain-specific knowledge. The weights of these identified features are increased by a random number in the interval [0.0,1.0) in the entire initial population, thereby giving them a higher weightage as compared to other features from the very beginning.

### 4.3. Mean-Based Crossover (MBC)

**Figure 2:**
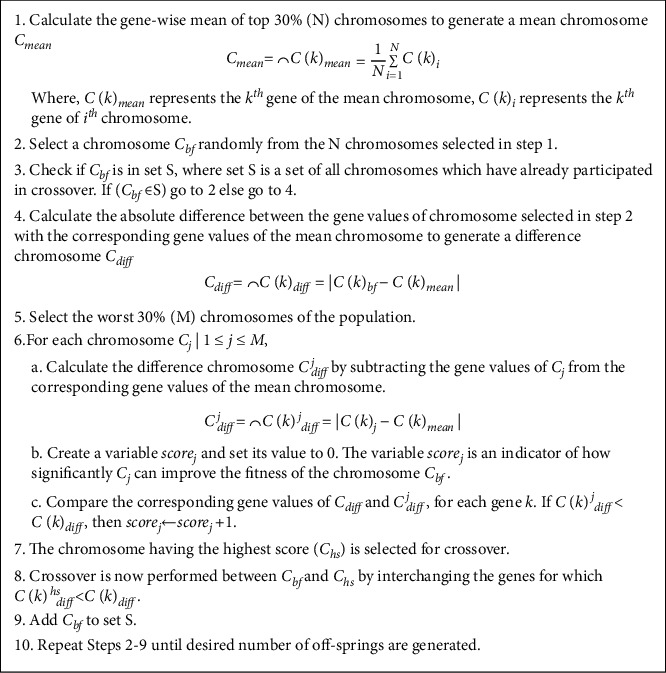
Pseudocode for MBC.

After population initialization, the next step is the application of various operators such as selection, crossover, and mutation repeatedly to obtain an MLP with optimal weights and biases. Maintaining diversity is important, but sometimes it is also vital to retain the best individuals of one generation into the next. GGA-MLP uses elitism to the transfer best chromosome(s) from one generation to another. Crossover and mutation are performed to generate offspring by selecting chromosomes from the current generation. The crossover operator takes two chromosomes and combines them to produce new offsprings. It is based on the idea that the exchange of information between good chromosomes will generate even better offsprings. Extreme care should be taken while performing selection and crossover operation as it may reduce the genetic diversity, which may ultimately lead to premature convergence. To avoid premature convergence, we present a crossover technique, known as mean-based crossover (MBC), that aims at improving the fitness of the top individuals of the population with the help of the worst members of the population. The proposed crossover technique involves the calculation of the mean of the fittest chromosomes in the population, thereby generating offsprings that are closer to the solution having minimum losses. Before applying MBC, GGA-MLP sorts the chromosomes in ascending order based on their fitness values. MBC starts by selecting the top 30% of the chromosomes and calculates the gene-wise mean of these chromosomes. The mean chromosome *C*_*mean*_ is an indicator of the ideal gene value which minimizes the MSE. In order to move toward a global optimum, this mean chromosome is used as a comparison parameter against individuals having low fitness values in the population. From the top 30% chromosomes, a chromosome *C*_*bf*_ is selected randomly for crossover. Another parent *C*_*hs*_ for crossover is chosen from the worst 30% individuals in such a way that it can contribute the most toward the fitness of chromosome *C*_*bf*_. The method of selection of *C*_*hs*_ is shown in Figure 2. After selecting *C*_*bf*_ and *C*_*hs*_, MBC is performed by exchanging the genes of *C*_*bf*_ and *C*_*hs*_ as shown in Figure 2. Out of the two children obtained from MBC, the offspring having higher fitness improves the quality of the population. The other offspring adds randomness to the population, thereby decreasing the probability of the population converging to a local optimum.

After crossover, *C*_*bf*_ is inserted into a set S to prevent it from being selected again for MBC in the current iteration. This is done to ensure that a unique chromosome is selected from the population each time MBC is performed, thereby preventing the problem of generating duplicate children. This process continues till the desired number of offsprings is generated. The steps of MBC are shown in Figure 2.

### 4.4. Greedy Mutation

In GA, the mutation operator is vital for maintaining diversity in the population. Mutation operator introduces diversity in the evolving population. It randomly modifies one or more genes of a chromosome depending upon the mutation probability which avoids getting stuck in the local minima. In traditional GA, every chromosome has an equal probability of getting mutated irrespective of its fitness [60]. It means both the best and the worst chromosomes have an equal probability of getting disrupted by mutation. In this paper, we propose a greedy mutation that aims to (i) avoid disruption of good quality chromosomes and (ii) at the same time maintain diversity in the population by mutating low-quality chromosomes, thereby improving the quality of the overall population.

Greedy mutation starts by calculating the gene-wise mean of the top 30% (N) chromosomes to generate a mean chromosome *C*_*mean*_. It then selects a chromosome *C*_*j*_ randomly from the worst 30% (*M*) chromosomes in the population for mutation. A random number *R* is generated for every gene of *C*_*j*_ and is compared with the mutation probability *P*_*m*_. If *R* >  *P*_*m*_, difference “*d*” between the value of the selected gene of *C*_*j*_ and that of the corresponding gene of the mean chromosome is calculated, and a random number “*r*” is generated. The product of *r* and *d* is then subtracted from the corresponding gene value in *C*_*j*_. This helps the chromosome in approaching good gene values, thereby increasing its overall fitness.

Due to the use of greedy approaches at each step, diversity of the population may decrease leading to premature convergence. To avoid this, it is important to introduce diversity in the population. GGA-MLP introduces diversity in the population in each iteration by generating 30% of the population using elitism, 50% of the population using MBC and greedy mutation, and the remaining 20% randomly by choosing synaptic weights and biases within the range [−2, 2].

## 5. Results and Discussion

First, we present the datasets that are selected to evaluate the effectiveness of GGA-MLP, in terms of accuracy achieved in classifying the input data, in Section 5.1. The implementation details, experimental setup used for performing experiments, and results are presented in Section 5.2.

### 5.1. Datasets

To evaluate the effectiveness of the proposed approach GGA-MLP, ten standard binary classification datasets are selected from the UCI Machine Learning Repository [61]: Parkinson, Indian Liver Patient Dataset (ILPD), Diabetes, Vertebral Column, Spambase, QSAR Biodegradation, Blood Transfusion, HTRU2, Drug Consumption: Amyl Nitrite, and Drug Consumption: Ketamine. The description of the selected datasets is shown in Table 1. In each dataset, 80% of the instances are used for training (out of which 20% is used for validation), and the remaining 20% are used for testing. It can easily be seen from Table 1 that the selected datasets have different numbers of features ranging from 4 to 57 as well as instances ranging from 197 to 17898, which helps us to evaluate the proposed approach on datasets of varying complexities. It also makes the task of evaluating GGA-MLP even more challenging.

### 5.2. Experimental Design and Results

To evaluate the effectiveness of GGA-MLP, the performance of MLP trained using GGA-MLP is compared with the classification accuracy of MLP trained using existing algorithms, namely, GA [21], ABC [27], MMPSO [28], WOA [33], MVO [34], and GOA [35], and on each dataset given in Table 1. All the algorithms are implemented in Python 3.6.4 using the Anaconda framework. As these are randomized algorithms, 30 runs of each algorithm are performed on every dataset. After each run, the best MLP is selected, and its classification accuracy on the test dataset is calculated using

(5) $$\begin{array}{c} Classification\;Accuracy=\frac{NC}{N}\text{,} \end{array}$$

where NC is the number of correctly classified testing data samples and *N* is the total number of samples in the testing dataset.

Before the start of the training phase, it is required to decide the architecture of MLP for each dataset. To perform a fair comparison, the architecture of MLP is kept the same for each algorithm. Here, we take only one hidden layer, as one hidden layer is sufficient to classify the datasets shown in Table 1. The number of neurons in the hidden layer is decided by using the method proposed by [25]. The number of neurons in the hidden layer is calculated using the formula 2 × *N*+1, where N is number of relevant features of the dataset. In some cases, the number of hidden neurons taken is 5 × *N*+1. The architecture of MLP used for each dataset is shown in Table 2.

The values of the controlling parameters of ABC, WOA, MMPSO, MVO, GOA, GA, and GGA-MLP are listed in Table 3. Various performance metrics such as classification accuracy, specificity, and sensitivity are used to assess the performance of GGA-MLP with respect to the existing state-of-the-art algorithms. The average, best, and standard deviation of classification accuracy, specificity, and sensitivity of the best MLP trained using these metaheuristic algorithms during 30 runs for the given datasets are shown in Tables 4–6, respectively. Data is collected under Windows 10 on Intel core i5-7200U 3.1 GHz processor with 8.00 GB DDR4 and Nvidia GT 940MX 2 GB VRAM.

It is evident from Tables 4 and 5 that GGA-MLP gives the highest average and best accuracy as well as specificity for the datasets except Parkinson, QSAR Biodegradation, Drug Consumption: Amyl Nitrite, and Drug Consumption: Ketamine. Despite having low accuracies and specificity on the four datasets, GGA-MLP achieves higher sensitivity as compared to the existing algorithms, as evident from Table 6, which shows the superiority of GGA-MLP in classifying the positive samples correctly. GGA-MLP also has low standard deviation as compared to existing state-of-the-art algorithms. This shows the robustness of the proposed approach.

**Figure 3:**
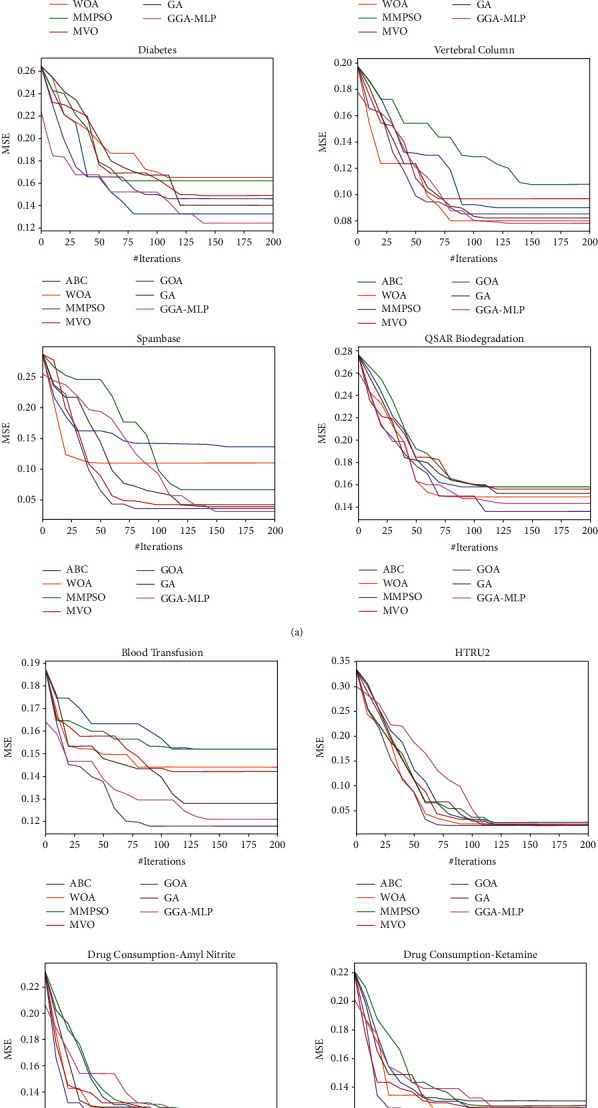
Convergence graph of MSE of MLP trained using ABC, WOA, MMPSO, MVO, GOA, GA, and GGA-MLP for 10 classification datasets.

In Figure 3, MSE values of MLP trained using ABC, WOA, MMPSO, MVO, GOA, GA, and GGA-MLP for the given datasets are calculated at an interval of 10 iterations and plotted to visualize convergence rate. The convergence curves show that although GGA-MLP takes more time to converge as compared to other metaheuristic algorithms, it avoids getting trapped in local minima. In most of the cases, the performance of GGA-MLP is better than the existing algorithms. To assess the efficacy of MLP trained using GGA-MLP as a classifier, we compare the classification accuracy of GGA-MLP with that of the classifiers built using other machine learning algorithms such as logistic regression, Naïve Bayes, and decision tree, as well as the MLP trained using BP. Similar to decision tree algorithms, BP algorithms like GGA-MLP are also randomized algorithms; every dataset is run 30 times on each of them, and the average, best, and standard deviation of classification accuracy are reported in Table 7. To prevent overfitting, validation set is used for early stopping during training of logistic regression, Naïve Bayes, and decision tree as well as the MLP using BP. It is clear from Table 7 that GGA-MLP gives the best result in all the cases. However, the standard deviation over 30 runs is least in case of decision tree. From Tables 4–7, it is clear that GGA-MLP performance is better than or comparable to the existing algorithms in classifying input patterns correctly.

## 6. Conclusion and Future Work

In this paper, a greedy genetic algorithm, GGA-MLP, is presented to train MLP. The use of domain-specific knowledge enables the generation of good quality initial population. Mean-based crossover and greedy mutation help algorithm in moving toward global optima by exploring the search space thoroughly. Datasets of varying complexities are used to evaluate the performance of GGA-MLP and to compare it with existing state-of-the-art algorithms as well as existing classifiers such as Naïve Bayes, decision tree, logistic regression, and MLP trained using BP. The results show that although GGA-MLP takes more time to converge as compared to other metaheuristic algorithms, the performance of GGA-MLP is better than or comparable to the existing techniques in classifying datasets, especially large datasets, as GGA-MLP searches the solution space properly by maintaining a balance between exploration and exploitation.

In future, we plan to extend our work to train other types of ANNs and incorporate architecture optimization in it.

## Figures and Tables

**Table 1:** Binary classification dataset.

#	Dataset	# features	# instances	# training instances	# testing instances
1	Parkinson	22	195	158	37
2	ILPD	5	583	467	116
3	Diabetes	8	768	615	153
4	Vertebral Column	6	310	248	62
5	Spambase	57	4601	3681	920
6	QSAR Biodegradation	41	1055	844	211
7	Blood Transfusion	4	748	598	150
8	HTRU2	8	17898	14318	3580
9	Drug Consumption: Amyl Nitrite	12	1885	1508	377
10	Drug Consumption: Ketamine	12	1885	1508	377

**Table 2:** MLP architecture and selected attributes for each dataset.

#	Dataset	MLP architecture (input-hidden-output)	Selected attributes for greedy population initialization
1	Parkinson	22-111-1	Average vocal fundamental frequency, minimum vocal fundamental frequency, maximum vocal fundamental frequency
2	ILPD	5-26-1	SGPT alanine aminotransferase, SGOT aspartate aminotransferase, direct bilirubin
3	Diabetes	8-17-1	Blood pressure, insulin, BMI
4	Vertebral Column	6-31-1	Lumbar lordosis angle, sacral slope, pelvic radius
5	Spambase	57-115-1	Length of the longest uninterrupted sequence of capital letters, average length of uninterrupted sequences of capital letters, total number of capital letters in the e-mail
6	QSAR Biodegradation	41-83-1	SpMax_L: leading eigenvalue from Laplace matrix, J_Dz(e): Balaban-like index from Barysz matrix weighted by Sanderson electronegativity
7	Blood Transfusion	4-9-1	R (recency - months since last donation), F (frequency - total number of donation), M (monetary - total blood donated in c.c.)
8	HTRU2	8-17-1	Mean of the DM-SNR curve, standard deviation of the DM-SNR curve, excess kurtosis of the DM-SNR curve, skewness of the DM-SNR curve
9	Drug Consumption: Amyl Nitrite	12-25-1	Nscore, Escore, Oscore, Ascore, Cscore
10	Drug Consumption: Ketamine	12-25-1	Nscore, Escore, Oscore, Ascore, Cscore

**Table 3:** Controlling parameters of metaheuristic algorithms.

Optimization algorithm	Parameter	Value
GGA-MLP, GA, ABC, WOA, MMPSO, MVO, GOA	Initial population size	30
	# iterations	200

GA, ABC, WOA, MMPSO, MVO, GOA	Initial population generation	Random population initialization (synaptic weights and biases are initialized randomly in the range [−2, 2])

GGA-MLP	Initial population generation	Greedy population initialization

GA, GGA-MLP	Probability of crossover (*P*_*c*_)	0.8
	Probability of mutation (*P*_*m*_)	0.05
	Elitism	30%

ABC	Random number (*ɸ*)	[−1, 1]

WOA	Vector $\left(\overset{⟶}{a}\right)$	Linearly decreasing from 2 to 0
	Random vector $\left(\overset{⟶}{r}\right)$	[0, 1]
	Constant (*b*)	1
	Random number (*p*)	[0, 1]
	Random number (*l*)	[−1, 1]

MMPSO	Acceleration coefficients (*c*_1_, *c*_2_)	1.48
	Inertia weights (*w*)	0.729
	Number of swarms	3
	Swarm size	10

MVO	Minimum wormhole existence probability	0.2
	Minimum wormhole existence probability	1

GOA	*c*_*max*_	1
	*c*_*min*_	0.00001
	L	1.5
	F	0.5

**Table 4:** Comparison results of classification accuracy of MLP trained using ABC, WOA, MMPSO, MVO, GOA, GA, and GGA-MLP.

Dataset\algorithm		ABC [27]	WOA [33]	MMPSO [28]	MVO [34]	GOA [35]	GA [21]	GGA-MLP
Parkinson	Avg	0.8042	0.7538	0.7481	0.9023	0.9294	0.8209	0.8666
	Std	0.062	0.0955	0.0609	0.0667	0.0531	0.0670	0.0772
	Best	0.9189	0.8649	0.8378	0.9459	0.9730	0.8919	0.9189

ILPD	Avg	0.6991	0.7009	0.7006	0.7000	0.7141	0.7018	0.7196
	Std	0.0047	0.0000	0.0016	0.0021	0.0201	0.0208	0.0302
	Best	0.7009	0.7009	0.7009	0.7094	0.7607	0.7265	0.7863

Diabetes	Avg	0.7416	0.7246	0.6647	0.7568	0.7576	0.7563	0.7697
	Std	0.0317	0.0296	0.0615	0.0263	0.0285	0.0267	0.0255
	Best	0.8039	0.7516	0.7647	0.7843	0.7778	0.7974	0.8301

Vertebral Column	Avg	0.8376	0.8504	0.7954	0.8453	0.8631	0.8317	0.8851
	Std	0.0632	0.0472	0.0639	0.0389	0.0353	0.0467	0.0365
	Best	0.8871	0.9032	0.8387	0.8710	0.8710	0.9032	0.9355

Spambase	Avg	0.7639	0.7491	0.8202	0.8432	0.8564	0.8465	0.9176
	Std	0.0295	0.0409	0.0279	0.0327	0.0239	0.0214	0.0176
	Best	0.7935	0.8152	0.8533	0.8761	0.9120	0.9022	0.9370

QSAR Biodegradation	Avg	0.7166	0.7329	0.7216	0.7564	0.8656	0.8049	0.8498
	Std	0.0698	0.0777	0.0563	0.0538	0.0302	0.0336	0.0422
	Best	0.8104	0.8246	0.7867	0.8104	0.9052	0.8483	0.8957

Blood Transfusion	Avg	0.7613	0.7800	0.7736	0.7976	0.8345	0.8342	0.8479
	Std	0.03503	0.00526	0.0210	0.005	0.0045	0.0059	0.0039
	Best	0.7800	0.7867	0.7933	0.8133	0.8533	0.8400	0.8667

HTRU2	Avg	0.9454	0.9678	0.9747	0.9656	0.9786	0.9799	0.9805
	Std	0.0225	0.0037	0.0015	0.0014	0.0022	0.0012	0.0010
	Best	0.9701	0.9763	0.9779	0.9712	0.9810	0.9816	0.9827

Drug Consumption: Amyl Nitrite	Avg	0.8298	0.8363	0.8359	0.8168	0.8242	0.8198	0.8356
	Std	0.0219	0.0013	0.0087	0.0202	0.0410	0.0113	0.0012
	Best	0.8435	0.8488	0.8462	0.8329	0.8541	0.8355	0.8462

Drug Consumption: Ketamine	Avg	0.8243	0.8216	0.8221	0.8032	0.8268	0.8111	0.8204
	Std	0.0162	0.0121	0.0129	0.0264	0.0301	0.0119	0.0116
	Best	0.8462	0.8435	0.8462	0.8276	0.8541	0.8276	0.8382

**Table 5:** Comparison results of specificity of MLP trained using ABC, WOA, MMPSO, MVO, GOA, GA, and GGA-MLP.

Dataset\algorithm		ABC [27]	WOA [33]	MMPSO [28]	MVO [34]	GOA [35]	GA [21]	GGA-MLP
Parkinson	Avg	0.7914	0.6734	0.7583	0.8460	0.8126	0.7865	0.7914
	Std	0.0805	0.0845	0.0632	0.0668	0.0798	0.0521	0.0601
	Best	0.9310	0.8276	0.8621	0.9655	0.9655	0.8621	0.8966

ILPD	Avg	0.4570	0.4222	0.4565	0.4126	0.4218	0.3546	0.4788
	Std	0.0064	0.0000	0.0031	0.0040	0.0083	0.0092	0.001
	Best	0.4889	0.4222	0.4667	0.4222	0.4444	0.3778	0.4889

Diabetes	Avg	0.6934	0.6521	0.5987	0.6965	0.7012	0.7051	0.7124
	Std	0.0621	0.0264	0.0743	0.0086	0.0043	0.0218	0.0723
	Best	0.7670	0.6990	0.7184	0.7184	0.7184	0.7476	0.7767

Vertebral Column	Avg	0.8026	0.8126	0.7962	0.8264	0.8556	0.8432	0.8643
	Std	0.0756	0.0316	0.0528	0.0254	0.0404	0.031	0.0264
	Best	0.8889	0.8519	0.8519	0.8519	0.8889	0.8889	0.8889

Spambase	Avg	0.6653	0.6521	0.7587	0.7865	0.7917	0.7664	0.8942
	Std	0.0085	0.0321	0.0262	0.0322	0.0853	0.0912	0.0010
	Best	0.7040	0.7291	0.7943	0.8227	0.8763	0.8629	0.9064

QSAR Biodegradation	Avg	0.6514	0.6954	0.7256	0.7381	0.8216	0.7021	0.8126
	Std	0.0954	0.0732	0.0063	0.0057	0.0156	0.0378	0.0054
	Best	0.7826	0.7899	0.7681	0.7681	0.8768	0.7826	0.8478

Blood Transfusion	Avg	0.7265	0.7355	0.7543	0.7632	0.8123	0.8011	0.8268
	Std	0.0765	0.0032	0.0082	0.0028	0.0014	0.0032	0.0021
	Best	0.7653	0.7551	0.7959	0.7857	0.8265	0.8265	0.8367

HTRU2	Avg	0.9452	0.9542	0.9702	0.9678	0.9721	0.9724	0.9742
	Std	0.0031	0.0026	0.0016	0.0015	0.0018	0.0015	0.0014
	Best	0.9724	0.9771	0.9802	0.9712	0.9814	0.9820	0.9824

Drug Consumption: Amyl Nitrite	Avg	0.8165	0.8321	0.8256	0.7982	0.7920	0.7814	0.7925
	Std	0.0076	0.0021	0.0062	0.0075	0.0168	0.0062	0.0047
	Best	0.8517	0.8486	0.8454	0.8265	0.8423	0.8297	0.8297

Drug Consumption: Ketamine	Avg	0.8945	0.9207	0.9112	0.8765	0.8965	0.8643	0.8621
	Std	0.0322	0.0051	0.0443	0.0556	0.0065	0.0234	0.0186
	Best	0.9366	0.9437	0.9401	0.9120	0.9190	0.8944	0.8944

**Table 6:** Comparison results of sensitivity of MLP trained using ABC, WOA, MMPSO, MVO, GOA, GA, and GGA-MLP.

Dataset\algorithm		ABC [27]	WOA [33]	MMPSO [28]	MVO [34]	GOA [35]	GA [21]	GGA-MLP
Parkinson	Avg	0.0864	0.9821	0.0715	0.8476	0.9820	0.9776	0.9845
	Std	0.0028	0.0032	0.0072	0.0068	0.0032	0.0036	0.0021
	Best	0.875	1.0000	0.7500	0.875	1.0000	1.0000	1.0000

ILPD	Avg	0.7945	0.8520	0.8025	0.8768	0.9327	0.9122	0.9543
	Std	0.0094	0.0046	0.0069	0.0038	0.0029	0.0037	0.0025
	Best	0.8333	0.8750	0.8472	0.8889	0.9583	0.9444	0.9722

Diabetes	Avg	0.8356	0.8432	0.7965	0.8976	0.8898	0.8657	0.9316
	Std	0.0412	0.0142	0.0684	0.0002	0.0002	0.0053	0.0001
	Best	0.8800	0.8600	0.8600	0.9200	0.9000	0.9000	0.94

Vertebral Column	Avg	0.8256	0.9206	0.7681	0.8542	0.8321	0.8765	0.9543
	Std	0.0209	0.0051	0.0564	0.0034	0.0032	0.0026	0.0010
	Best	0.8857	0.9429	0.8286	0.8857	0.8571	0.9143	0.9714

Spambase	Avg	0.8675	0.9342	0.9125	0.9430	0.9532	0.9355	0.9785
	Std	0.0622	0.0332	0.0204	0.0031	0.0025	0.0078	0.0021
	Best	0.9596	0.9752	0.9627	0.9752	0.9783	0.9752	0.9938

QSAR Biodegradation	Avg	0.7217	0.8536	0.7621	0.8765	0.9332	0.9452	0.9720
	Std	0.0721	0.0501	0.0420	0.0028	0.0024	0.0032	0.0001
	Best	0.8630	0.8904	0.8219	0.8904	0.9589	0.9726	0.9863

Blood Transfusion	Avg	0.8575	0.8921	0.8543	0.8965	0.9329	0.9056	0.9486
	Std	0.0022	0.0002	0.0036	0.0003	0.0002	0.0004	0.0002
	Best	0.8824	0.9024	0.8764	0.9167	0.9419	0.9205	0.9535

HTRU2	Avg	0.8970	0.9432	0.9123	0.9547	0.9589	0.9546	0.9698
	Std	0.0107	0.0037	0.0068	0.0041	0.0028	0.0030	0.0026
	Best	0.9486	0.9686	0.9571	0.9714	0.9771	0.9771	0.9857

Drug Consumption: Amyl Nitrite	Avg	0.7886	0.8222	0.7986	0.8443	0.9065	0.8245	0.9124
	Std	0.0310	0.0230	0.0865	0.0020	0.0003	0.0048	0.0022
	Best	0.80	0.85	0.85	0.8667	0.9167	0.8667	0.9333

Drug Consumption: Ketamine	Avg	0.7589	0.7234	0.7308	0.7765	0.9010	0.8438	0.9216
	Std	0.0076	0.0037	0.0083	0.0045	0.0037	0.0043	0.0010
	Best	0.8030	0.7576	0.7879	0.8030	0.9242	0.8788	0.9394

**Table 7:** Comparison of performances of classifiers.

Dataset\algorithm		Logistic regression	Naïve Bayes	Decision tree	BP	GGA-MLP
Parkinson	Avg	0.8205	0.6154	0.8162	0.8496	0.8666
	Std	—	—	0.0316	0.0089	0.0772
	Best	0.8205	0.6154	0.8718	0.8718	0.9189

ILPD	Avg	0.7094	0.5128	0.6786	0.7179	0.7196
	Std	—	—	0.0203	—	0.0302
	Best	0.7094	0.5128	0.7179	0.7179	0.7863

Diabetes	Avg	0.7403	0.7597	0.6541	0.7558	0.7697
	Std			0.0113	0.0122	0.0255
	Best	0.7403	0.7597	0.6818	0.7857	0.8301

Vertebral Column	Avg	0.8065	0.7097	0.6398	0.8500	0.8851
	Std	—	—	0.0191	0.0128	0.0365
	Best	0.8065	0.7097	0.6774	0.8871	0.9355

Spambase	Avg	0.9054	0.8239	0.9092	0.9241	0.9176
	Std	—	—	0.0040	0.0022	0.0176
	Best	0.9054	0.8239	0.9185	0.9283	0.9370

QSAR Biodegradation	Avg	0.8436	0.6682	0.8052	0.8561	0.8498
	Std	—	—	0.0107	0.0105	0.0422
	Best	0.8436	0.6682	0.8246	0.8720	0.8957

Blood Transfusion	Avg	0.7867	0.7733	0.8267	0.7600	0.8479
	Std	—	—	—	0.0063	0.0039
	Best	0.7867	0.7733	0.8267	0.7733	0.8667

HTRU2	Avg	0.9765	0.9411	0.9624	0.9703	0.9805
	Std	—	—	0.0013	0.0014	0.0010
	Best	0.9765	0.9411	0.9645	0.9724	0.9827

Drug Consumption: Amyl Nitrite	Avg	0.7931	0.6976	0.6943	0.7926	0.8356
	Std	—	—	0.0088	0.0098	0.0012
	Best	0.7931	0.6976	0.7135	0.8090	0.8462

Drug Consumption: Ketamine	Avg	0.7931	0.7454	0.7080	0.7724	0.8204
	Std	—	—	0.0243	0.0216	0.0116
	Best	0.7931	0.7454	0.7268	0.8011	0.8382

## Data Availability

No data were used to support this study.
